# Constant-Beamwidth Beamforming with Concentric Ring Arrays

**DOI:** 10.3390/s21217253

**Published:** 2021-10-31

**Authors:** Avital Kleiman, Israel Cohen, Baruch Berdugo

**Affiliations:** Andrew and Erna Viterby Faculty of Electrical and Computer Engineering, Technion—Israel Institute of Technology, Technion City, Haifa 3200003, Israel; saviklei@campus.technion.ac.il (A.K.); bbaruch@technion.ac.il (B.B.)

**Keywords:** array processing, constant-beamwidth beamformer, circular sensor array, concentric ring array

## Abstract

Designing beampatterns with constant beamwidth over a wide range of frequencies is useful in many applications in speech, radar, sonar and communication. In this paper, we design constant-beamwidth beamformers for concentric ring arrays. The proposed beamformers utilize the circular geometry to provide improved beamwidth consistency compared to beamformers which are designed for linear sensor arrays of the same order. In the proposed configuration, all sensors on each ring share the same weight value. This constraint significantly simplifies the beamformers and reduces the hardware and computational resources required in a physical setup. Furthermore, a theoretical justification of the beamforming method is provided. We demonstrate the advantages of the proposed beamformers compared to the one-dimensional configuration in terms of directivity index, white noise gain and sidelobe attenuation.

## 1. Introduction

Applications in communication, radar and speech processing require dealing with broadband signals [1]. A wide variety of tasks, such as source separation, noise reduction and signal enhancement, are enhanced by using wideband beamforming algorithms in these applications [2,3]. To avoid non-uniform attenuation and distortion caused by the beamformer, several approaches implementing frequency invariant beamformers were presented in the literature [4,5,6,7,8,9,10,11,12]. Implementing the desired beampattern is commonly done by changing the weights given to each sensor under specified restrictions.

One of the main challenges associated with designing a wideband beamformer is maintaining a constant beamwidth over a wide range of frequencies. In common beamforming methods (as delay and sum [13]), the mainbeam becomes narrower as the frequency increases. Many constant-beamwidth beamformers can be applied to produce beampatterns with better beamwidth consistency and lower sensitivity. Beamforming algorithms using least-squares, linearly constrained minimum variance, adaptive delay lines and minimum variance distortionless response were suggested in the literature [14,15,16,17,18]. However, some of these approaches suffer from high computational complexity and degradation in performance measures (such as sensitivities to model mismatches, diverseness of sensors, and deviation from nominal configuration).

Our design follows the approach proposed by Rosen et al. [19]. This method demonstrated low computational complexity as well as improved array response results in various scenarios, compared to other methods in the field. The proposed approach utilizes custom-tailored filters for each sensor, manipulating the beampattern beamwidth to remain constant. The basic idea of the suggested algorithm includes a gradual elimination of active sensors to achieve the desired constant beamwidth in different frequencies. The elimination of the sensors is done by attenuating their signals as the frequency increases. This changes the effective aperture of the array and results in beamwidth consistency. According to this approach, each sensor output is processed by a lowpass filter with different cutoff frequencies, depending on the sensor position. In between the cutoff frequencies, smoothing magnitude coefficients are calculated such that the beamwidth remains constant. In addition, a post-summation normalization is applied to the output result, so the gain at all the frequencies is also constant. This method showed better sidelobe attenuation and robustness to array mismatches compared to other methods.

Most of the work on constant-beamwidth beamformers, and specifically the beamformer mentioned above, deals with a 1D uniform linear array (ULA) geometry. Yet, there is a growing interest in using 2D array geometries, specifically circular arrays, in beamforming algorithms. Utilizing the uniform circular array (UCA) symmetry has the potential of superior performance in terms of noise suppression, direction of arrival (DOA) estimation and design flexibility. Hence, in applications where the signal of interest may come from any direction, circular arrays are advantageous [20,21,22].

Due to the progress in audio and communication applications, many beamforming methods were proposed for uniform concentric circular arrays (UCCAs) over the last decade. A classical criterion in synthesizing the desired beampattern is minimizing the minimum mean-square-error (MMSE). This approach for UCCAs was suggested in [23], and was later incorporated in [24,25] as an optimization cost function. The employment of convex optimization to attain broadband fixed pattern was also suggested in [26], formulating a second-order cone programming problem. In [27], this method was modified to 3D spherical arrays. Recently, the use of differential sensor arrays on the circular geometry was investigated in [28,29,30,31,32]. The suggested beamformers provided enhanced performance in terms of steering flexibility and beampatterns irregularities. In addition, in [33], a greedy-based sparse design was introduced, optimizing simultaneously the number of rings and the number of sensors in the array. This approach was later generalized in [34], focusing on the 2D scenario. Window-based beamformers were also presented in the literature for the task of designing frequency-invariant beampatterns. In [35], a Kaiser window was applied on the UCCA to control the sidelobe levels while attaining the required beamwidth. The window-based approach in [36,37] was used to weight the sensors on the rings. The suggested method enabled a control of the elevation and azimuth beamwidth. Most of the presented UCCA beamformers consider the scenario in which the signal-of-interest arrives from the horizontal plane. However, in practical 3D applications the elevation angle is not confined to be $90^{\circ }$. The arbitrary elevation angle directly affects the directivity factor (DF) and the white noise gain (WNG), which are important performance measures of the array.

In this paper, we present beamformers with constant elevation beamwidth, covering a pre-defined area of interest. The pre-configured locations of the sensors in the array eliminate the need of beam steering. The weight given to each sensor is derived from theoretical considerations. The exact weight value is calculated to attain the desired beamwidth while gradually eliminating sensors from outer rings. In the design process, we utilize concentric ring arrays (CRAs). That is, we restrict all the sensors on each ring to have the same weights. This restriction allows a significant improvement in computational complexity, since the number of weights is determined by the number of rings in the array, rather than the number of sensors. In addition, designing joint weights to all the sensors on a given ring enables an analog summation of the sensors’ signals before sampling. Accordingly, this design enables to use a single A/D sampler per ring, instead of sampling each sensor individually. We introduce a design method in the low frequency range that modifies the filters applied to each array element from lowpass filters to bandpass filters. This method exploits the circular geometry and results in better performance compared to beamformers that are designed for ULA with an equivalent number of channels (i.e., beamformer order).

The paper is organized as follows: Section 2 describes the motivation behind the suggested design. We describe the advantages of the proposed approach compared to existing methods. In Section 3, we formulate the problem, and describe the geometrical setup. Section 4 provides a theoretical justification of implementing our constant-beamwidth design on circular geometries. Section 5 introduces the design method of the constant-beamwidth CRA, including a particular consideration to low frequencies and a time domain implementation of the desired beamformer filters. Section 6 evaluates the performance of the proposed beamformer compared to a constant-beamwidth beamformer, of the same order, that is designed for ULAs. Finally, some conclusions are drawn in Section 7.

## 2. Motivation

Frequency invariant beamformers are used, e.g., in multi-speakers settings, such as large conference rooms, auditoriums and lecture halls. The problem considered in our paper deals with designing circular ceiling array beamformers, covering multiple beamforming zones.

There are several types of beamforming technologies for designing ceiling sensor arrays. Dynamic beamforming approach adaptively steers the mainbeam to the desired speaker direction. The steering is preformed by separately sampling each sensor element and applying individual complex weight to each sensor. While there are advantages to adaptive beamforming, such as flexibility in the speaker locations, it usually requires more hardware resources or higher computational complexity. In addition, adaptive beamformers are used to steer the mainbeam to focus on the current speaker. Hence, the performance of such beamformers in practical environments depends on the direct-to-reverberant (DRR) ratio and the speakers’ distance from the array [38,39,40]. Moreover, planar arrays are usually constructed of a large number of elements. A multichannel beamforming design that steers the beam adaptively requires a significant number of phase shifters, amplifiers, samplers and filters. The physical hardware affects the array cost, maintenance, sensitivities to model mismatches, diversity of sensors, and deviation from nominal configuration [41].

**Figure 1:**
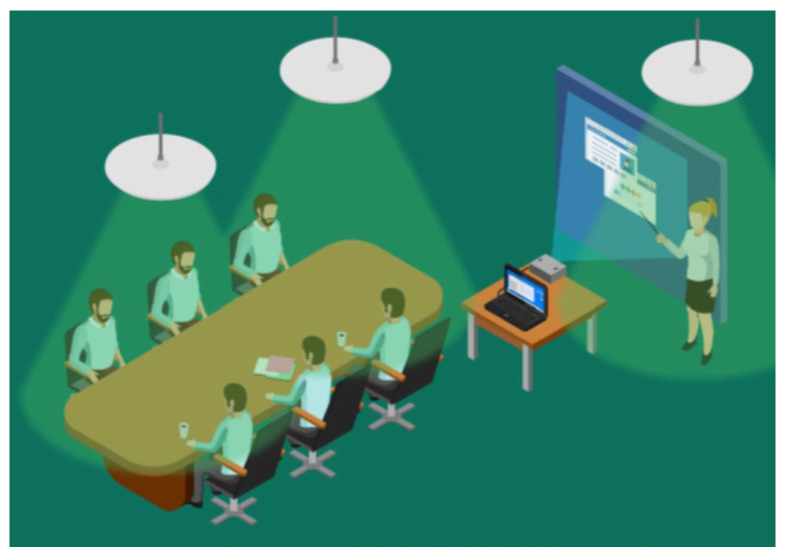
Conference room illustration with circular ceiling array covering multiple beamforming zones. Illustration courtesy of Stem Audio.

Developing ceiling sensor arrays with multiple fixed beam-forming zones that incorporate predetermined recording regions is a way of eliminating the need for beam steering. The proposed system considered in our paper is illustrated in Figure 1. Given a room layout and a number of ceiling circular arrays, one can cover the area of interest by combining several fixed beamformers with designated beamwidth. Each signal in the scope of the main beam arrives to the closest array from broadside with a certain elevation angle. Afterwards, the signal is processed with the beamformer weights that are calculated as described in the paper. A system of this type offers the following benefits:

(1) **Fixed beam direction**: as the source signal is assumed to be within the area covered by the main lobe, there is no need to estimate the source location and steer the beam to the estimated direction.
(2) **Low resource setup:** by eliminating the need of steering the beam, the proposed design enables to use fewer A/D samplers, phase shifters and filters, which is significant in 2D arrays. In the proposed method, all sensors on each ring share joint weights. As a result, we allow an analog summation of all the received signals from a given ring before sampling.
(3) **Real-time processing:** since the number of channels in each array is determined by the number of rings (rather than the number of sensors), the beamformer includes less filters, has lower computational complexity, and processing time is faster.

We introduce a constant-beamwidth beamformer that can be incorporated in the multi-beam system described above. We present a simple, low resource design of the beamformer weights and explain the physical considerations of choosing the array radii and number of sensors.

## 3. Problem Formulation

**Figure 2:**
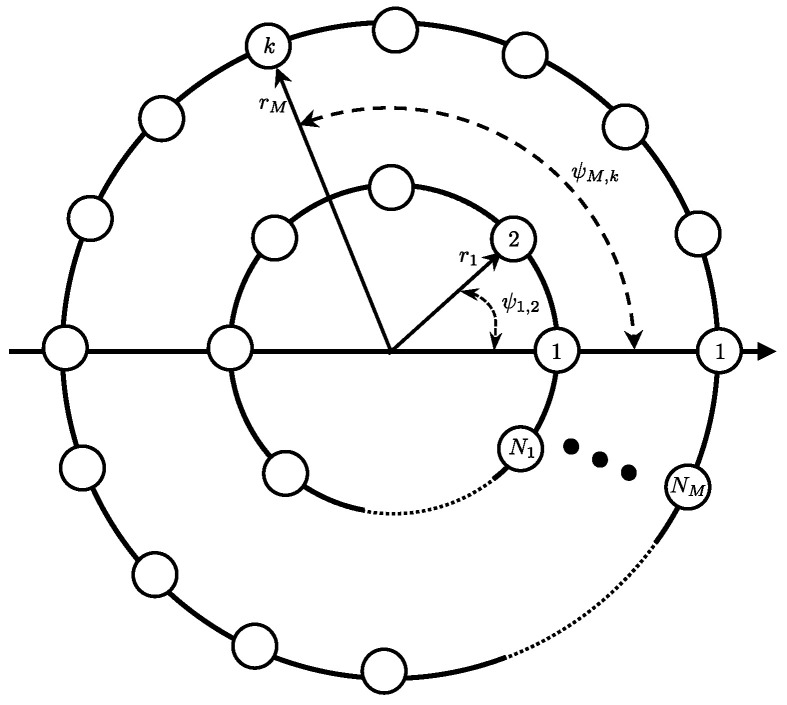
Illustration of a CRA with *M* rings, where $\psi_{m,k}$ indicates the angular position of the *k*th element on the *m*th ring, constructed of $N_{m}$ sensor elements.

In this section, we describe a discrete CRA, constructed of equally spaced omnidirectional sensor elements on each ring. We consider a CRA composed of *M* rings, containing $N_{m}$$\left(m=1,2,\ldots ,M\right)$ sensors in each ring, and receiving a source signal in the farfield (steered to broadside) as shown in Figure 2.

Given a ring with radius $r_{m}$, the placement of the *k*th sensor $\left(k\in [1,N_{m}]\right)$ on a Cartesian coordinate system is given by:

(1) $$\begin{array}{c} r_{m,k}=r_{m}\left(\cos\psi_{m,k},\sin\psi_{m,k},0\right)\;,\; \end{array}$$

where $\psi_{m,k}$ is the angle of the *k*th sensor on the *m*th ring, measured anti-clockwise from the x-axis:

(2) $$\begin{array}{c} \psi_{m,k}=\frac{2\pi \left(k-1\right)}{N_{m}}\;. \end{array}$$

We consider a plane wave with a wave vector $\alpha \overset{▵}{=}\left[\sin\theta \cos\phi ,\sin\phi \sin\theta ,\cos\theta \right]$, where ϕ is the azimuth angle and θ is the elevation angle. The time delay between the origin of the circular array and the signal received at the *k*th sensor on the *m*th ring is

(3) $$\begin{array}{c} \tau_{m,k}=\frac{\alpha \cdot r_{m,k}}{c}=\frac{r_{m}\cos\left(\phi -\psi_{m,k}\right)\sin\theta }{c}\;, \end{array}$$

where c is the signal propagation speed (i.e., 340m/s for acoustic signals propagating in the air). Having a broadband signal $x\left(t\right)$ received in the origin point, the *k*th sensor element output is

(4) $$\begin{array}{c} y_{m,k}\left(t\right)=x\left(t-\tau_{m,k}\right)\;. \end{array}$$

In the frequency domain, the signal model is given by

(5) $$\begin{array}{c} Y_{m,k}\left(f\right)=e^{-j2\pi f\tau_{m,k}}X\left(f\right)\;, \end{array}$$

where *f* is the temporal frequency. Collecting all the outputs of the sensors on the *m*th ring, we obtain

(6) $$\begin{array}{c} {\underline{y}}_{m}=\left[Y_{m,1}\left(f\right),Y_{m,2}\left(f\right),\ldots ,Y_{m,N_{m}\left(f\right)}\right]=X\left(f\right){\underline{d}}_{m}\left(f,\theta ,\phi \right)\;,\; \end{array}$$

where ${\underline{d}}_{m}\left(f,\theta ,\phi \right)$ is the steering vector of the *m*th ring:

(7) $$\begin{array}{cc} {\underline{d}}_{m}\left(f,\theta ,\phi \right) & \overset{▵}{=}\left[\begin{array}{c} e^{j\frac{2\pi r_{m}f}{c}\cos\left(\phi -\psi_{m,1}\right)\sin\theta }\\ e^{j\frac{2\pi r_{m}f}{c}\cos\left(\phi -\psi_{m,2}\right)\sin\theta }\\ \vdots \\ e^{j\frac{2\pi r_{m}f}{c}\cos\left(\phi -\psi_{m,N_{m}}\right)\sin\theta } \end{array}\right]. \end{array}$$

By concatenating the *M* steering vectors, we express the array steering vector as:

(8) $$\begin{array}{c} \underline{d}\left(f,\theta ,\phi \right)={\left[{\underline{d}}_{1}^{T}\left(f,\theta ,\phi \right),ߪ,{\underline{d}}_{M}^{T}\left(f,\theta ,\phi \right)\right]}^{T}\; \end{array}$$

where ${}^{T}$ denotes the transpose operator.

**Figure 3:**
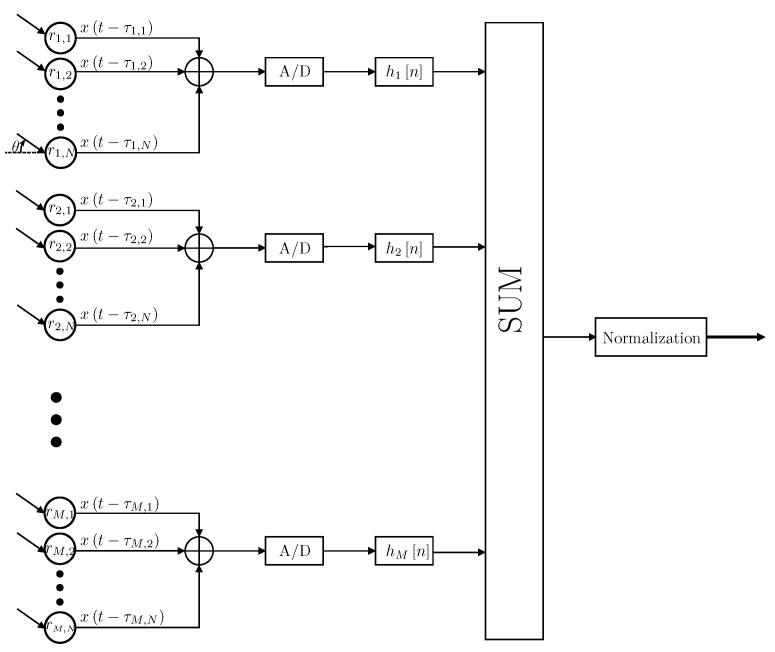
Block diagram of joint weights CRA beamformer.

In the proposed beamformer design, all the sensors in the *m*th ring are multiplied by the same weight value (in a given frequency *f*), denoted by $H_{m}\left(f\right)$. Designing the beampattern under this constraint provides two main advantages in practical applications. First, the computational complexity of designing the beamformer weights is significantly lower. Second, designing joint weights to all the sensors on a given ring enables an analog summation of the sensors’ signals before sampling. In physical systems, this design enables the use of a single A/D sampler per ring, instead of sampling each sensor signal individually, which simplifies the required hardware. A block diagram of the proposed design is provided in Figure 3.

A source signal arriving in angle θ to the circular array is delayed by $\tau_{m,k}$ according to the sensor placements. The output signals are summed per-ring and sampled. Afterwards, each ring response is filtered by the temporal filters denoted by $h_{m}\left[n\right]$, where $m\in \left[1,M\right]$. The filter results are later summed and normalized by normalization filter to yield the output signal.

Given this design restriction, the weigh vector of the *m*th ring is given by:

(9) $$\begin{array}{c} {\underline{h}}_{m}\left(f\right)=H_{m}\left(f\right){\underline{1}}_{1\times N_{m}}\;, \end{array}$$

where **${\underline{1}}_{1\times N_{m}}$** is the unit vector of length $N_{m}$. Let $N\overset{▵}{=}\sum_{m=1}^{M}N_{m}$ denote the total number of sensors in the array. Concatenating all the weight vectors into an $\left(N\times 1\right)$ vector, representing the weights applied on the array elements at frequency *f*, we obtain

(10) $$\begin{array}{c} \underline{h}\left(f\right)={\left[{\underline{h}}_{1}\left(f\right),...,{\underline{h}}_{M}\left(f\right)\right]}^{T}\;. \end{array}$$

The CRA beampattern is defined as the summation of all the weighted outputs signals, i.e.,

(11) $$\begin{array}{c} B\left[\underline{h}\left(f\right),\theta ,\phi \right]={\underline{h}}^{T}\left(f\right)\underline{d}\left(f,\theta \right)=\sum_{m=1}^{M}H_{m}\left(f\right)\sum_{k=1}^{N_{m}}e^{j\frac{2\pi r_{m}f}{c}\cos\left(\phi -\psi_{m,k}\right)\sin\theta }\;. \end{array}$$

It can be shown that with a sufficient number of sensors, the beampattern of the discrete sensor ring is approximately equal to the continuous ring beampattern [42]. As a result, we can assume that the beampattern is independent of the azimuth angle ϕ. Hence, without loss of generality, we can choose $\phi \overset{▵}{=}0$, implying that

(12) $$\begin{array}{c} B\left[\underline{h}\left(f\right),\theta \right]\approx \sum_{m=1}^{M}H_{m}\left(f\right)\sum_{k=1}^{N_{m}}e^{j\frac{2\pi r_{m}f}{c}\cos\psi_{m,k}\sin\theta }\;. \end{array}$$

To simplify the notation, we denote the discrete ring response $R_{m}\left(f,\theta \right)$ as

(13) $$R_{m}\left(f,\theta \right)\overset{▵}{=}\sum_{k=1}^{N_{m}}e^{j\frac{2\pi r_{m}f}{c}\cos\psi_{m,k}\sin\theta }\;.$$

## 4. Theoretical Analysis

In order to design a constant-beamwidth beampattern for CRA, our suggested beamformer includes a gradual elimination of the outer rings in the array as the frequency increases. In this section, we provide a theoretical justification to the proposed approach by analyzing the nature of the beampattern for continuous CRA geometry.

Let us first examine the beampattern of a rigid plane piston which is dependent on the temporal frequency *f*, the piston radius *R* and the signal elevation angle θ as follows [43]

(14) $$\begin{array}{c} B_{p}\left(f,R,\theta \right)=\frac{J_{1}\left(\frac{2\pi f}{c}R\sin\theta \right)}{\frac{\pi f}{c}R\sin\theta }\;, \end{array}$$

where $J_{1}$ is the first-order Bessel function. Note that due to the symmetry of the problem, the beampattern does not depend on the azimuth angle.

Let $\theta_{\mathrm{BW}}$ define the desired beamwidth, achieved in the −3 dB amplitude of the beampattern and denoted as $\beta_{\mathrm{BW}}\overset{▵}{=}0.7$. The constant-beamwidth constraint in a given frequency range defined by $\left[f_{L},f_{H}\right]$ implies that:

(15) $$\begin{array}{c} \forall f\in \left[f_{L},f_{H}\right]:\;\;B_{p}\left(f,R,\frac{\theta_{\mathrm{BW}}}{2}\right)=\beta_{\mathrm{BW}}\;. \end{array}$$

Using the beampattern defined in (14), we obtain

(16) $$\begin{array}{c} \frac{2\pi f}{c}R\sin\left(\frac{\theta_{\mathrm{BW}}}{2}\right)\approx 1.638\;. \end{array}$$

Since we aim to keep the beamwidth constant, the value of $\sin\left(\frac{\theta_{\mathrm{BW}}}{2}\right)$ should not change over a wide range of frequencies. This can be achieved if the value of the product fR is kept constant. Hence, decreasing the piston radius as the frequency increases yields a constant beamwidth.

The CRA geometry contains concentric rings, which effectively sample the piston in discrete radii. The beampattern of a continuous circular ring laying in the x-y plane is:

(17) $$\begin{array}{c} B_{r}\left(f,r,\theta \right)=J_{0}\left(\frac{2\pi f}{c}r\sin\theta \right)\;, \end{array}$$

where $J_{0}$ is the zero-order Bessel function and *r* is the ring radius [43]. Note that similarly to (16), the beamwidth of the beampattern in (17) depends on the frequency and ring radius. Hence, as seen in the piston beampattern, to achieve a constant beamwidth, we need to establish a constant fr relation.

Given a CRA constructed from *M* concentric rings, the beampattern will be a weighted sum of the beampatterns shown in (17):

(18) $$\begin{array}{c} B\left(f,\underline{r},\theta \right)=\sum_{m=1}^{M}H_{m}\left(f\right)J_{0}\left(\frac{2\pi f}{c}r_{m}\sin\theta \right)\;, \end{array}$$

where $H_{m}\left(f\right)$ denotes the weight given to the *m*-th ring in the frequency *f* and $\underline{r}\overset{▵}{=}\left[r_{1},\ldots ,r_{M}\right]$ is the radii vector of the CRA rings. By changing the weights given to each ring as the frequency increases, we can control the effective radius of the sampled piston such that the beamwidth will remain constant, as discussed above. In the following section, the beamformers’ design is presented, relying on the radius–frequency relation with a gradual decrease of the effective radius.

## 5. CRA Constant-Beamwidth Beampattern Design

As discussed in Section 4, the beamwidth of the CRA remains constant if we ensure that the radius–frequency product is constant. Thus, in this section, we suggest a beamformer design that involves elimination of the outer rings in the CRA as the frequency increases. We also explain the geometric considerations in choosing the number of sensors constructing the array.

All the results shown in this section and in Section 6 are for a CRA constructed of M=6 rings with radii $\underline{r}=\left[2.5,5,10,15,20,25\right]\;\mathrm{cm}$ having 16 sensors each and a desired beamwidth of **$\theta_{\mathrm{BW}}=30{}^{\circ }$**. Note that even though the array contains a large number of elements, the number of weights calculated per frequency is determined by the number of rings. For simplicity, the shown results are for a broadside array.

### 5.1. Number of Sensors

As mentioned in Section 3, the array is constructed of equally spaced discrete sensor elements. To avoid spatial aliasing, the spacing between the elements should be smaller than half of the wavelength λ [43]. Assuming that the sensor placement is uniform on each ring, the inner spacing should satisfy:

(19) $$\begin{array}{c} \delta_{m}=2r_{m}\sin\left(\frac{\pi }{N_{m}}\right)\approx \frac{2\pi r_{m}}{N_{m}}<\frac{c}{2f}\;, \end{array}$$

where $\delta_{m}$ is the inner space between two adjacent sensors. Let $r_{\mathrm{out}}$ denote the radius of the outermost effective ring, satisfying $H_{\mathrm{out}}\left(f\right)\neq 0$. From (19), the number of sensors on the outer ring in a given frequency *f* should comply with

(20) $$\begin{array}{c} N_{\mathrm{out}}>\frac{4\pi r_{\mathrm{out}}f}{c}\;. \end{array}$$

Since we attenuate the outer rings as the frequency increases, $r_{\mathrm{out}}$ is smaller for higher frequencies. Thus, the relation $r_{\mathrm{out}}f$ remains constant. Consequently, the right side of the inequality is constant for a wide range of frequencies. This implies that the number of elements $N_{\mathrm{out}}$ should not change when decreasing the effective radius. As a result, the number of sensors should be constant for all the rings in the CRA.

Equation (16) presents the relation between the radius–frequency product and the desired beamwidth for a rigid piston beampattern:

(21) $$fR\approx \frac{1.638c}{2\pi \sin\left(\frac{\theta_{\mathrm{BW}}}{2}\right)}.$$

Substituting fR into (20) yields the following dependency between the number of sensors and the desired beamwidth

(22) $$N>\frac{4\pi }{c}\frac{1.638c}{2\pi \sin\left(\frac{\theta_{\mathrm{BW}}}{2}\right)}=\frac{3.276}{\sin\left(\frac{\theta_{\mathrm{BW}}}{2}\right)}.$$

Under the chosen beamwidth restriction, we have N>12.7. To verify that the chosen number of discrete sensor elements in the CRA is sufficient, we examine the discretization error between the continuous ring CRA beampattern and the discrete CRA in the following manner:

(23) $$\begin{array}{c} \varepsilon =\max_{f,\theta }\left|\sum_{m=1}^{M}H_{m}\left(f\right)\left[R_{m}\left(f,\theta \right)-J_{0}\left(\frac{2\pi f}{c}r_{m}\sin\theta \right)\right]\right|\;, \end{array}$$

where f∈[0.9,8]kHz and $\theta \in [-60^{\circ },60^{\circ }]$. The evaluation of the maximal error in (23) for different numbers of discrete sensors shows that having 16 elements on each ring results in a ∼$10^{-6}$ difference between the expected and the achieved beampattern.

### 5.2. Calculating the Attenuation Coefficients

The goal of the presented step is to determine the attenuation weights of the beamformer as the frequency increases. In this stage, all the rings are effective in the beamforming process, so that the weight given to each ring is equal to one. As frequency increases, the outer rings are attenuated until eventually only one ring contributes to the beamforming process. This gradual elimination process is similar to sampling a piston with a smaller radius for higher frequencies. As explained in Section 4, this approach keeps the value of fR constant, resulting in constant beamwidth.

To find the frequency range in which the desired beamwidth remains constant, let us define the lowest and highest frequencies $f_{L}$ and $f_{H}$ for which the beamwidth is attained for a given CRA. These frequency bounds are determined by two weight combinations: when all the rings are active, i.e., $H_{m}\left(f_{L}\right)=1\;\;\forall m\in \left[1,M\right]$, the lower bound of the frequency range satisfies

(24) $$\begin{array}{c} \beta_{\mathrm{BW}}=\frac{1}{N}\sum_{m=1}^{M}R_{m}\left(f_{L},\frac{\theta_{\mathrm{BW}}}{2}\right)\;. \end{array}$$

When only the innermost ring is active, i.e., $H_{1}\left(f_{H}\right)=1$ and $H_{m}\left(f_{H}\right)=0$ for m≠1, the upper bound of the frequency range satisfies

(25) $$\begin{array}{c} \beta_{\mathrm{BW}}=\frac{1}{N_{1}}R_{1}\left(f_{H},\frac{\theta_{\mathrm{BW}}}{2}\right)\;. \end{array}$$

To ensure that the beampattern is equal to 1 at boresight, we normalize it by the number of active sensors in the beamforming.

Since (24) and (25) are difficult to solve analytically, we use the bisection method to find the roots of the above equations in the range $f\in \left[0,10\right]\;\mathrm{kHz}$. The bisection method yields $f_{L}=1617\;\mathrm{Hz}$ and $f_{H}=9405\;\mathrm{Hz}$ (we limit the maximal frequency to 8000Hz). By repeating this process for all *M* possible array radii constellations (i.e., when all rings are active, the M−1 inner rings are active, etc.), we find the *M* transition frequencies where the outermost ring is fully attenuated. Given a frequency in the range $f\in \left[f_{L},f_{H}\right]$, we can design the beamformer to achieve $\theta_{\mathrm{BW}}$, as described next.

We define $f_{m}$ as the frequency obtaining the desired beamwidth in an *m* rings array (where 0<m<M). We denote the total number of sensors in an *m* rings CRA as ${\hat{N}}_{m}\overset{▵}{=}\sum_{k=1}^{m}N_{k}$. Given a frequency in the transition band $f\in \left[f_{m+1},f_{m}\right]$, the *m* inner rings are active and the m+1 ring is attenuated such that:

(26) $$\begin{array}{c} H_{i}\left(f\right)=\left\{\begin{array}{cc} 1, & i\in \left[1,m\right],\\ \in \left(0,1\right), & i=m+1. \end{array}\right. \end{array}$$

To obtain the desired beamwidth, the exact weight value, applied on the outer m+1 ring, satisfies

(27) $$\begin{array}{c} \beta_{\mathrm{BW}}=\frac{\sum_{i=1}^{m}R_{i}\left(f,\frac{\theta_{\mathrm{BW}}}{2}\right)+H_{m+1}\left(f\right)R_{m+1}\left(f,\frac{\theta_{\mathrm{BW}}}{2}\right)}{{\hat{N}}_{m}+H_{m+1}\left(f\right)N_{m+1}}\;. \end{array}$$

Some mathematical manipulations of (27) lead to the attenuation amplitude of the outer ring:

(28) $$\begin{array}{c} H_{m+1}\left(f\right)=\frac{\beta_{\mathrm{BW}}{\hat{N}}_{m}-\sum_{i=1}^{m}R_{i}\left(f,\frac{\theta_{\mathrm{BW}}}{2}\right)}{R_{m+1}\left(f,\frac{\theta_{\mathrm{BW}}}{2}\right)-\beta_{\mathrm{BW}}N_{m+1}}\;. \end{array}$$

Hence, the weight value for the *m*th ring ($m\in \left[1,M\right]$) is given by

(29) $$\begin{array}{c} H_{m}\left(f\right)=\left\{\begin{array}{cc} 1, & f<f_{m},\\ \frac{\beta_{\mathrm{BW}}{\hat{N}}_{m-1}-\sum_{i=1}^{m-1}R_{i}\left(f,\frac{\theta_{\mathrm{BW}}}{2}\right)}{R_{m}\left(f,\frac{\theta_{\mathrm{BW}}}{2}\right)-\beta_{\mathrm{BW}}N_{m}}, & f_{m}<f<f_{m-1},\\ 0, & f_{m-1}<f. \end{array}\right. \end{array}$$

**Figure 4:**
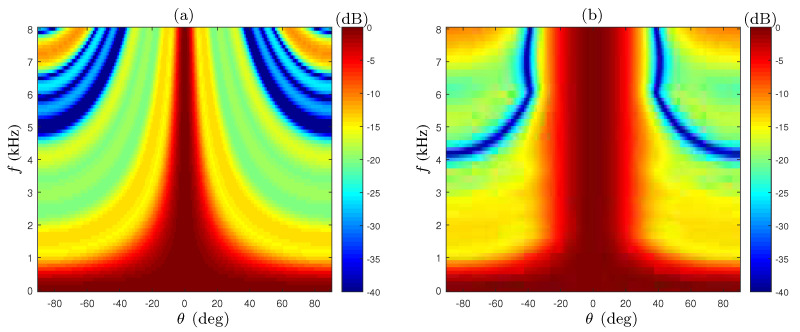
Beampatterns of the 6-ring CRA, (**a**) without constant-beamwidth beamformer weights, and (**b**) after applying the constant-beamwidth beamformer weights.

The array beampattern of the lowpass filters beamformer (designed according to (29)) and without them (i.e., the result of a simple summation) are shown in Figure 4. We see that in the frequency range $f>f_{L}$, the mainlobe width after applying the filters (plot (b)) remains constant, as opposed to the changing beamwidth shown in Figure 4a. However, in the lower frequency range $f<f_{L}$, the main lobe is wider as the frequency decreases in both cases. The next step of the suggested beamformer aims to extend the frequency range of the constant beamwidth. In the suggested approach, the array filters are designed as bandpass filters, instead of lowpass filters, by utilizing the circular geometry.

### 5.3. Weights Adaptation in Low Frequencies

We aim to extend the frequency range for which the beampattern attains the desired beamwidth. To do so, we examine the beampattern of a single ring constructed of discrete sensor elements. As mentioned in Section 3, the beampattern of the discrete ring is approximately equal to the continuous beampattern if a sufficient number of sensors is chosen. Hence, the beampattern of the discrete ring is similar to the zero-order Bessel function of the first kind, shown in (17).

In the first beamformer design step, we assumed that all the rings are active in low frequencies, resulting in lowpass filters applied on the CRA. As a result, the lowest frequency for which the desired beamwidth is attained depends on the piston beampattern shown in (14). However, the beampattern of a single ring allows an extension of the low frequency range. By assuming that a single ring with radius *r* is active, we find that the beamwidth is satisfied if

(30) $$\begin{array}{c} \frac{2\pi f}{c}r\sin\left(\frac{\theta_{\mathrm{BW}}}{2}\right)\approx 1.41\;. \end{array}$$

From (30), we note that at frequencies lower than $f_{L}$, the beampattern can attain the desired beamwidth if we design the array to hold a single active ring. Therefore, we add the following step to the design process: At low frequencies, all the CRA weights are null except for the outer ring. As the frequency increases, we gradually add the inner rings while maintaining a constant beamwidth, until the frequency reaches the lower bound $f=f_{L}$. From that point, where all the rings are active in the beamformer, we repeat the gradual elimination of the outer ring as described in the previous section. Having the extended beamwidth approach, the new lower frequency bound is given by

(31) $$\begin{array}{c} \beta_{\mathrm{BW}}=\frac{1}{N_{M}}R_{M}\left(f_{L}^{\mathrm{new}},\frac{\theta_{\mathrm{BW}}}{2}\right)\;. \end{array}$$

Equation (31) is satisfied for $f_{L}^{\mathrm{new}}=940\;\mathrm{Hz}$, compared to the lower bound in the original implementation which was $f_{L}=1617\;\mathrm{Hz}$. By designing the filters as bandpass filters, we can extend the constant-beamwidth frequency range. To calculate the weights for the inner rings in the extended range $f\in \left[f_{L}^{\mathrm{new}},f_{L}\right]$, we repeat the bisection process and find the transition frequency bins.

In the suggested design, we denote $f_{m}$ as the frequency where the M−m outer rings are active in the beamformer and the *m*th inner ring is gradually enhanced. For $f\in \left[f_{m+1},f_{m}\right]$, the weight of the outer ring is $H_{i}\left(f\right)=1\;\forall i\in \left[m+1,M\right]$ while the inner *m*th ring is added to the array such that $H_{m}\in \left(0,1\right)$. Since we aim to increase the constant-beamwidth frequency range, the weight given to the inner ring satisfies

(32) $$\begin{array}{cc} \beta_{\mathrm{BW}} & =\frac{\sum_{i=m+1}^{M}R_{i}\left(f,\frac{\theta_{\mathrm{BW}}}{2}\right)+H_{m}\left(f\right)R_{m}\left(f,\frac{\theta_{\mathrm{BW}}}{2}\right)}{{\tilde{N}}_{m}+H_{m}\left(f\right)N_{m}}\;, \end{array}$$

where ${\tilde{N}}_{m}=\sum_{k=m+1}^{M}N_{k}$ is the total number of sensors in the active outer rings. From (32), the weight given to the inner ring in the lower frequency range is

(33) $$\begin{array}{c} H_{m}\left(f\right)=\frac{\beta_{\mathrm{BW}}{\tilde{N}}_{m}-\sum_{i=m+1}^{M}R_{i}\left(f,\frac{\theta_{\mathrm{BW}}}{2}\right)}{R_{m}\left(f,\frac{\theta_{\mathrm{BW}}}{2}\right)-\beta_{\mathrm{BW}}N_{m}}\;. \end{array}$$

**Figure 5:**
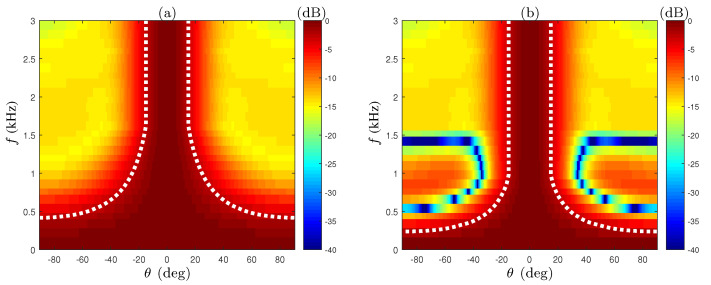
Beampatterns of the 6-ring CRA, (**a**) without lower frequencies beamwidth extension, and (**b**) with the extension. The dashed line marks the amplitude in which the BW is defined $\beta_{\mathrm{BW}}\overset{▵}{=}-3\;\mathrm{dB}$.

The beampattern of the extended beamwidth CRA (EXT-CRA) compared to the unmodified CRA in the low frequency range $f\in \left[0,3000\right]\;\mathrm{Hz}$ is shown in Figure 5. The gradual addition of the rings, starting from the outer ring inwards, provides better beamwidth consistency in frequencies smaller than $f_{L}$. For frequencies lower than 940Hz (for which only the outer ring is active), we cannot modify the beampattern, so the beamwidth is wider as the frequency decreases.

### 5.4. Directivity Index Improvement

In Section 5.3, the beamformer filters were reshaped to broaden the frequency range that attains the desired beamwidth. We can, however, improve the beamformer’s performance further by relaxing the beamwidth constraint at lower frequencies.

In the suggested step, the weights of the 2 outer rings are modified for frequencies $f\leq f_{L}^{\mathrm{new}}$ to maximize the directivity index (DI). The DI represents the array gain in a diffuse noise environment, and defined as [2]

(34) $$\begin{array}{c} D\left[\underline{h}\left(f\right)\right]\overset{▵}{=}\frac{{\left|\underline{h}{\left(f\right)}^{T}\underline{d}\left(f,\theta_{0},\phi_{0}\right)\right|}^{2}}{\underline{h}{\left(f\right)}^{T}\Gamma \underline{h}\left(f\right)}\;, \end{array}$$

where the pseudo-coherence matrix Γ is given by

(35) $$\begin{array}{c} \Gamma \overset{▵}{=}\frac{1}{4\pi }\int_{0}^{2\pi }\int_{0}^{\pi }\underline{d}\left(f,\theta ,\phi \right)\underline{d}{\left(f,\theta ,\phi \right)}^{T}\sin\theta d\theta d\phi \;. \end{array}$$

In order to improve the DI in low frequencies, we search for the optimal ratio between the outermost ring and the second outermost ring weights under the constraint that the beamwidth is wider than $\theta_{\mathrm{BW}}$. Hence, we can utilize the circular geometry in the lower frequency range to gain higher DI. A similar approach applied on one-dimensional ULA was suggested in [44]. The results of the proposed design compared to the previous stages are presented in Section 6.

### 5.5. Time Domain Filter Implementation

The previous sections presented the desired beamformer response given specified frequency characteristics. In this section, we determine the temporal FIR filter coefficients. The design aims to approximate the resulted beamformer response to the ideal beamformer described above.

The time-domain FIR filters are calculated from their frequency responses using the signal processing toolbox of MATLAB, which minimizes the integrated squared error defined as [45]:

(36) $$\begin{array}{c} \epsilon_{2}=\int_{0}^{\pi }W\left(\omega \right){\left(\hat{H}\left(\omega \right)-H\left(\omega \right)\right)}^{2}d\omega \;, \end{array}$$

where ω is the normalized frequency, $W\left(\omega \right)$ is the non-negative weighting function, $\hat{H}\left(\omega \right)$ is the actual filter response and $H\left(\omega \right)$ is the ideal response. The FIR filters are designed to have the same number of coefficients to maintain a fixed delay. The number of coefficients is chosen based on the mainbeam constraint [46]. The constraint ensures that the resulting beampattern at $\theta_{\mathrm{BW}}$ is approximately equal to $\beta_{\mathrm{BW}}$ over a grid of frequencies in the range $f_{i}\in \left[f_{L}^{\mathrm{new}},f_{H}\right]$:

(37) $$\begin{array}{c} \epsilon_{\mathrm{BW}}=\sum_{i}^{}{\left|B\left[\underline{h}\left(f_{i}\right),\theta_{\mathrm{BW}}\right]-\beta_{\mathrm{BW}}\right|}^{2}\Delta f\;. \end{array}$$

By iterating over a range of possible filter lengths, a 36-coefficient FIR filter per-ring was designed. This length compromises between the mainbeam constraint and the short delay requirement.

In addition, the beamformer should maintain a uniform gain over the whole frequency range to prevent distortion of the input signal. Hence, following the FIR filtering, the output results are summed and processed by an additional FIR normalization filter. The normalization filter taps are calculated by summing the absolute values of the previously calculated sensor filters and multiplied by the number of elements per-ring, as described in [19].

The entire design process is summarized in Algorithm 1. Note that even though the results shown in this section are for $\theta_{\mathrm{BW}}=30^{\circ }$, the proposed beamformer design supports significantly lower beamwidths. However, choosing a smaller beamwidth increases the minimal frequency for which the desired beamwidth is achieved as can be observed from (30). Moreover, a lower beamwidth may require more sensors on each ring, as obtained from (22).

**Figure 6:**
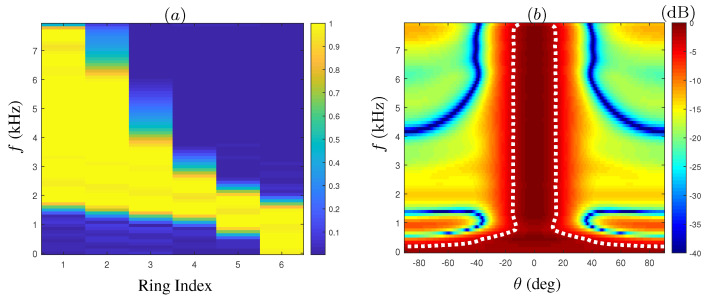
Constant-beamwidth beamformer for 6-ring CRA implemented with temporal FIR filters. (**a**) Weight values applied on the rings; (**b**) beampattern.

The weights matrix of the suggested beamformer is shown in Figure 6a. The y-axis represents frequency bins used for designing the array filters, and the x-axis represents the ring index (1–6), where index 1 is the innermost ring. Notice the gradual addition of inner rings in the lower frequency range, followed by the elimination of outer rings as the frequency increases. The colorbar indicates the attenuation weight value, ranging from 0 to 1. The final beampattern of the suggested beamformer is presented in Figure 6b. The −3dB amplitude is marked by the dashed white lines, which defines the beamwidth. We note that there is a slight degradation in the beamwidth consistency compared to the ideal beampattern, yet overall, the desired beamwidth is attained over a wide range of frequencies.
**Algorithm 1** Constant-Beamwidth CRA Beamformer Design*Calculate the attenuation weight:***for** $m\in \;\left[1,M\right]$ **do**   find $f_{m}$ by solving: $\beta_{\mathrm{BW}}=\frac{\sum_{i=1}^{m}R_{i}\left(f_{m},\frac{\theta_{\mathrm{BW}}}{2}\right)}{\sum_{i=1}^{m}N_{i}}$ using the Bisection algorithm.**end for**Define $f_{L}=f_{M}$ , $f_{H}=\min\left(f_{1},8000\right)$.**for **$f\in \;\left[f_{L},f_{H}\right]$** do**   Calculate ring weight based on (29).**end for***Modify the beamformer to expand the constant-beamwidth frequency range:***for** $m\in \;\left[1,M\right]$ **do**   find $f_{m}$ by solving: $\beta_{\mathrm{BW}}=\frac{\sum_{i=m}^{M}R_{i}\left(f_{m},\frac{\theta_{\mathrm{BW}}}{2}\right)}{\sum_{i=m}^{M}N_{i}}$ using the Bisection algorithm.**end for**Define $f_{L}^{\mathrm{new}}=f_{M}$.**for** $f\in \;\left[f_{L}^{\mathrm{new}},f_{L}\right]$ **do**   Compute the ring weight based on (33).**end for***Improve the DI in lower frequencies:***for** $f\in \;\left[0,f_{L}^{\mathrm{new}}\right]$ **do**   let $H_{M}\left(f\right)=1$, find $H_{M-1}\left(f\right)$ s.t $\underline{h}{\left(f\right)}^{T}\Gamma \underline{h}\left(f\right)$ is minimal.**end for***Implement the desired filters in time domain.**Calculate the normalization factor for each frequency.*

## 6. Performance Analysis

To evaluate the performance of the different beamformers discussed above, we use the DI and WNG measures. We also measure the first sidelobe level of the beampatterns and compare the beamwidth consistency throughout a range of frequencies and different beamwidths.

These performance measures are used to compare the following beamformers:

1. Constant-beamwidth ULA, which is designed using the algorithm suggested in [19], based on the symmetry around the center element. The length of the ULA equals to the diameter of the CRA, i.e., L=50cm. The element spacing is d=3.5cm to prevent spatial aliasing, and the ULA consists of 15 sensors;
2. A 6-ring CRA applied with the lowpass design (CRA-LP) (as described in Section 5.2);
3. A 6-ring CRA with the lower frequency beamwidth extension (EXT-CRA), suggested in Section 5.3;
4. The improved DI beamformer (EXT-CRA-I) in low frequencies by allowing a moderate widening of the beampattern. This design modification is described in Section 5.4.

All the performance measures presented in the following section were calculated after implementing each beamformer in the time domain, as described in Section 5.5. Note that even though the number of sensor elements in the CRA is larger compared to the ULA, the suggested CRA beamformer is composed of only 6 channels (equivalent to the number of rings in the array). Moreover, the beamformer is designed to attain constant elevation beamwidth with efficient application in a physical setup. As a result, the existing UCCA beamformers (mentioned in the introduction) which require considerably more physical equipment and computational resources are not presented in this section.

### 6.1. Beamwidth Consistency

**Figure 7:**
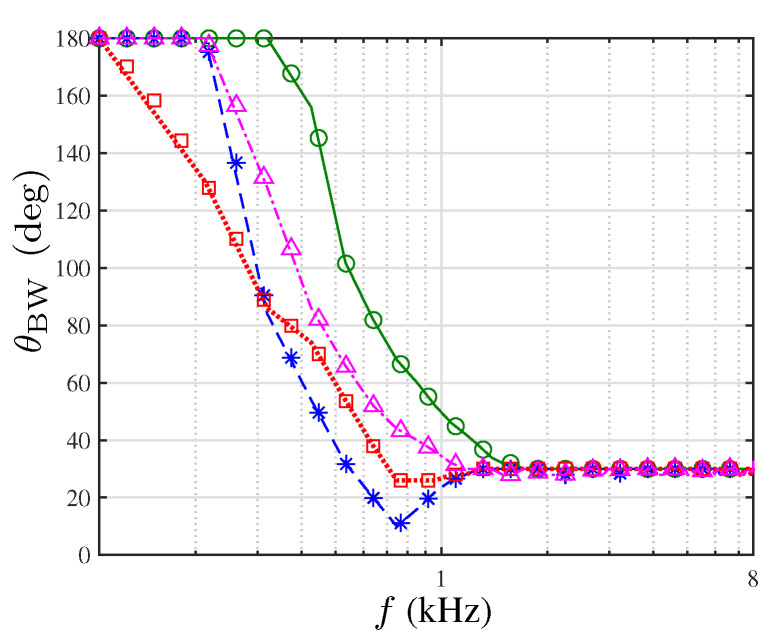
Beamwidth $\theta_{\mathrm{BW}}$ as a function of frequency for different beamformers: CRA-LP (solid line with circle), EXT-CRA (dashed line with asterisks), EXT-CRA-I (dotted line with squares) and ULA (dash-dotted line with triangles).

The main objective of the proposed beamformer is to design a constant-beamwidth beampattern over a wide range of frequencies. Figure 7 presents the beamwidth of each beamformer as a function of frequency. Compared to the CRA-LP design, the adaptation of the weights in lower frequency (EXT-CRA) results in smaller beamwidth in the frequencies $f\in \left[940,1617\right]\;\mathrm{Hz}$. Furthermore, for frequencies lower than 940Hz, the beamwidth increases as the frequency decreases for all the examined beamformers. However, the beamwidth after the suggested modification is smaller compared to CRA-LP and the ULA. In addition, we note that the DI improvement in the EXT-CRA-I beamformer affects the attained beamwidth compared to the EXT-CRA. Due to the time domain implementation, there is an inconsistency of the beamwidth in lower frequencies for the EXT-CRA beamformer. The EXT-CRA-I incorporates an additional ring in the beamforming process, which smoothes the ideal frequency response at lower frequencies. Hence, the frequency responses of the FIR filters are closer to the desired responses, which improves the beamwidth consistency in the lower frequency range.

### 6.2. Directivity Index and White Noise Gain

The WNG is a measure of the array gain in a spatially uncorrelated noise environment, which is a good indicator of the performance robustness to sensor imperfections. The WNG is calculated as [2]

(38) $$\begin{array}{c} W\left[\underline{h}\left(f\right)\right])=\frac{{\left|\underline{h}{\left(f\right)}^{T}\underline{d}\left(f,\theta_{0},\phi_{0}\right)\right|}^{2}}{\underline{h}{\left(f\right)}^{T}\underline{h}\left(f\right)}\;. \end{array}$$

**Figure 8:**
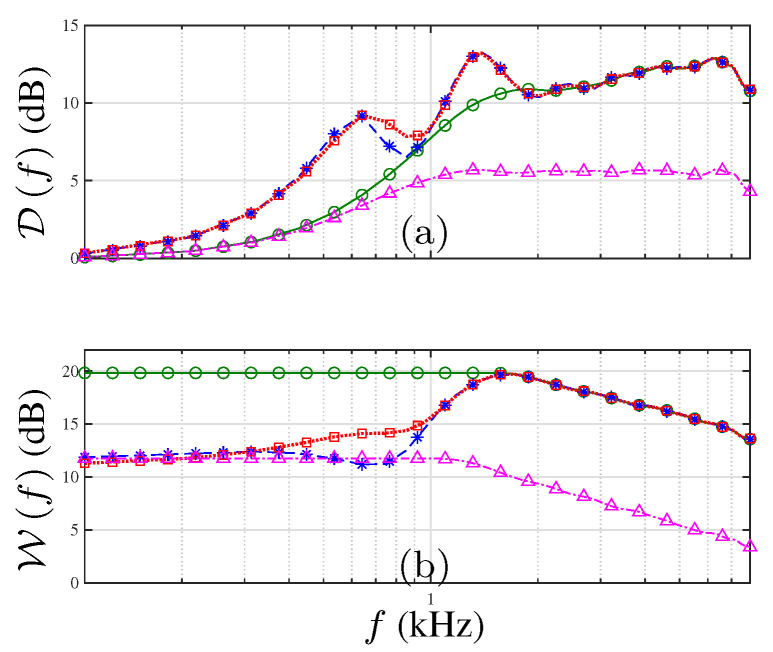
Performance measures for CRA-LP (solid line with circle), EXT-CRA (dashed line with asterisks), EXT-CRA-I (dotted line with squares) and ULA (dash-dotted line with triangles): (**a**) DI, and (**b**) WNG, as a function of frequency.

Figure 8 shows the performances of the discussed beamformers in terms of DI and WNG. Notice that the CRA geometry achieves higher DI compared to the ULA. In the frequency range f>1620Hz, the average difference is 7dB. Note the improvement in terms of DI between CRA-LP and the EXT-CRA beamformer in the lower frequency range. Furthermore, the EXT-CRA-I design achieves higher DI around f=846Hz compared to EXT-CRA, as desired.

The WNG comparison in Figure 8b shows that the WNG of the CRA is 10dB higher than the ULA for frequencies in the range f>1620Hz. Since the number of sensors in the LP-CRA is significantly higher than the number used in the ULA, it is more robust to incoherent noise due to electronic noise and errors in the sensor placing. Moreover, despite the improvements in beamwidth consistency, the WNG was smaller than the WNG achieved with the non-modified CRA beamformer. For frequencies $f<f_{L}$, there is a 8dB difference between the CRA-LP beamformer and the EXT-CRA beamformer.

### 6.3. Sidelobe Attenuation

**Figure 9:**
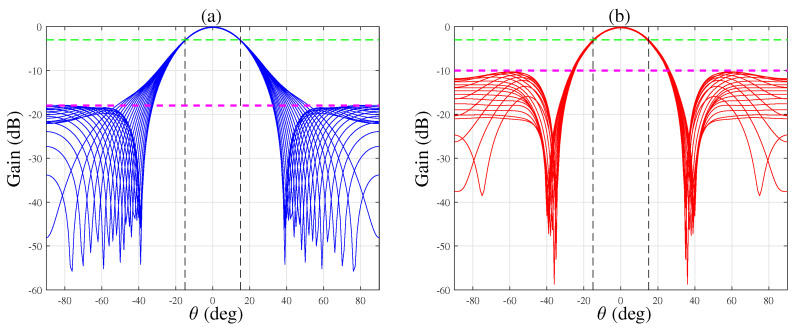
Beampatterns at different frequencies calculated for (**a**) EXT-CRA-I, and (**b**) ULA. The first sidelobe level, the desired BW and the −3 dB amplitude are marked by a dash-point line, solid line and dotted line, respectively.

The sidelobe attenuation can be helpful in attenuating signals from out-of-beamwidth directions and improving the directivity index. Figure 9 shows the beampatterns of the EXT-CRA-I and ULA beamformers calculated at different frequencies in the range $f\in \left[2000,8000\right]\;\mathrm{Hz}$. In terms of sidelobe attenuation, we see that the first sidelobe of the EXT-CRA-I beamformer is lower than the sidelobe of the ULA by 7dB. We observe that both arrays satisfy the beamwidth constraint.

### 6.4. Beamwidth Variability

**Figure 10:**
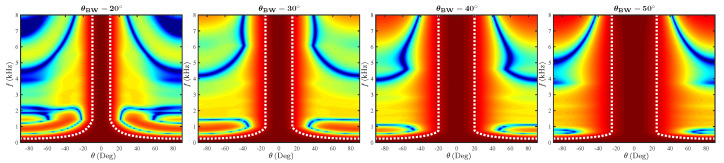
Beampatterns of a 6-ring CRA with several desired beamwidth specifications. The presented beampatterns are for $\theta_{\mathrm{BW}}=\left[20^{\circ },30^{\circ },40^{\circ },50^{\circ }\right]$, ordered from left to right.

An important characteristic of a constant-beamwidth beamformer is its ability to support variable system specifications. Thus, in this section, we present the beamformers’ performance for different desired beamwidths. That is, the beamformers’ competence to attain various beamwidths (under a constant ring constellation and number of sensors) is examined. Figure 10 shows beampatterns for $\theta_{\mathrm{BW}}=\left[20^{\circ },30^{\circ },40^{\circ },50^{\circ }\right]$ of the EXT-CRA beamformer. Note that under a similar geometry, the proposed beamformer attains multiple specified beamwidths. Marked by dashed-lines, the beamwidth remains consistent throughout a wide range of frequencies. As the required beamwidth is wider, the minimal frequency for which it is attained decreases. The relation between the lower bound frequency and the beamwidth was shown in (30). At lower frequencies, only the outermost ring is active while the rest of the rings are fully attenuated. Consequently, the radius is constant and the lower frequency bound is inversely dependent on the desired beamwidth.

Moreover, as the beamwidth is wider, the sidelobe levels are higher in high frequencies (f>6kHz). The attenuation of outer rings occurs in lower frequencies for wider beamwidths. As a result, as the frequency increases, the significant weight is applied on the innermost ring. Since the ring beampattern shares the characteristics of the zero-order Bessel function, the first sidelobe is closer as the frequency increases. Hence, as the inner ring is more dominant for a given wider beamwidth, the sidelobes are emphasized in higher frequencies.

**Figure 11:**
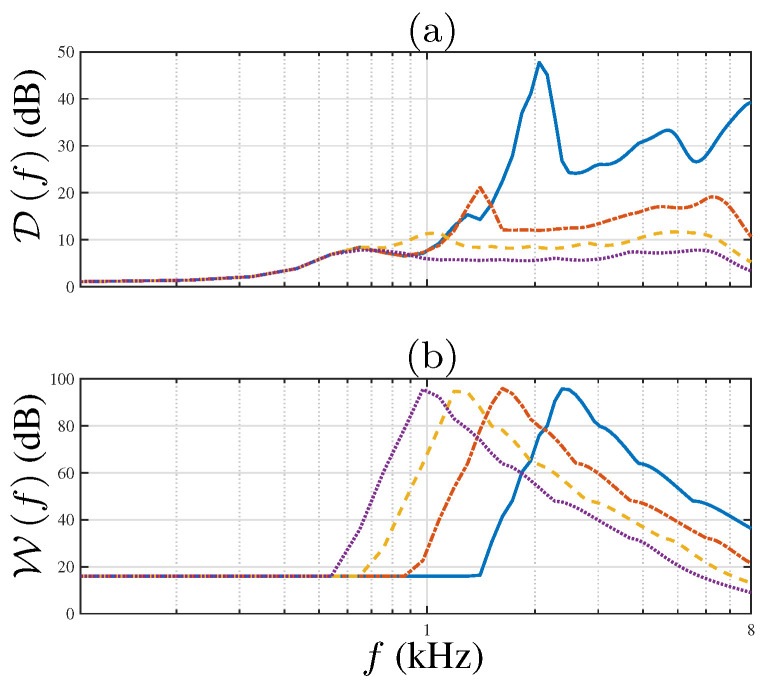
(**a**) DI, and (**b**) WNG, as a function of frequency, for EXT-CRA with beamwidths: $\theta_{\mathrm{BW}}=20^{\circ }$ (solid line), $\theta_{\mathrm{BW}}=30^{\circ }$ (point-dashed line), $\theta_{\mathrm{BW}}=40^{\circ }$ (dashed line) and $\theta_{\mathrm{BW}}=50^{\circ }$ (dotted line).

The beamwidth and the sidelobe level affect the beamformers’ performance. The DI and WNG for the different beamwidths are presented in Figure 11. As expected, the DI increases as the beamwidth is narrower. Note that the maximal DI for each beamwidth is obtained at lower frequencies for wider beamwidths. In addition, the decrease of the DI in higher frequencies is shown for $\theta_{\mathrm{BW}}=\left[30^{\circ },40^{\circ },50^{\circ }\right]$. The degradation is caused due to the sidelobe levels, as discussed above.

By examining the WNG plot, we note that as the beamwidth is wider, the increase and the following decrease of the WNG occurs at smaller frequencies. Since the beamwidth is achieved at lower frequencies, the gradual addition of inner rings increases the WNG. When all the rings in the CRA are active, the attenuation of the outer rings gradually reduces the WNG. As the number of sensors is equal per-ring, the WNG graphs present equivalent trend with shifting along the frequency axis.

## 7. Conclusions

We have introduced beamformers for concentric ring arrays, designed to have a constant beamwidth over a wide range of frequencies. The suggested beamformers are designed to be incorporated in a multi-beam system of circular ceiling arrays. We described the advantages of the discussed system and the specifications required from the beamformer. The proposed design applies the same filter to all the elements on each ring. This constraint lowers computational complexity in design and enables a physical setup that requires fewer resources. In addition, we modified the lower frequency band by reducing the number of active rings in that range. The suggested modification utilizes the geometrical properties of the array to extend the frequency range for which a constant beamwidth is achieved. We also added an improved directivity index design in frequencies lower than the range limits, and implemented the beamformer in the time domain with FIR filters. The proposed beamformer showed superior performance in terms of directivity index, white noise gain, beamwidth consistency and sidelobe attenuation, compared to a constant-beamwidth beamformer of the same order, designed for a ULA.

The beamformers in this work are designed for uniformly spaced rings. We also assumed that the elimination of rings as the frequency changes is continuous (from the outer ring inwards). However, it is possible that there is a way to utilize the Bessel properties in an optimal way to design a constant-beamwidth beampattern while improving other criteria. The variability in the sensor placements, the number of elements on the rings, and the relative positions of different rings can be investigated in future studies.
